# Pruritus: From the Bench to the Bedside

**DOI:** 10.1155/2018/5742753

**Published:** 2018-05-22

**Authors:** Adam Reich, Laurent Misery, Kenji Takamori

**Affiliations:** ^1^Department of Dermatology, University of Rzeszow, Rzeszow, Poland; ^2^Department of Dermatology, University Hospital Brest, Brest, France; ^3^Institute for Environmental and Gender Specific Medicine and Department of Dermatology, Juntendo University Graduate School of Medicine, Urayasu, Chiba, Japan

Pruritus is defined as a unpleasant sensation that causes a desire to scratch. It may accompany various skin diseases, but it may also be present in many systemic, neurologic, and psychiatric conditions. However, it is often overlooked or its significance is diminished. Remarkably, many studies documented that, for patients, pruritus is often considered as the most bothersome symptom in the course of the diseases they suffer from. Fortunately, the awareness about the relevance of pruritus is steadily growing among physicians and health care professionals. Subsequently, the higher awareness results in a growing interest in studying the underlying mechanisms of pruritus. Hopefully, our growing understanding of its pathogenesis will eventually generate new antipruritic treatment modalities and improve patient care in the near future. The current special issue is focusing on the latest developments in the field of pruritus science, providing new insights into pruritus pathogenesis, showing new data on pruritus clinical manifestation, and discussing new treatment options for pruritic conditions.

A paper by J. Song et al. could be handled as a nice introduction to the current special issue on pruritus, giving the readers an overview of the current understanding on the transmission of pruritic stimuli to the brain, describing the potential mediators of pruritus in the skin, including proteases, cytokines, peptides, and phospholipid metabolites, and explaining the differences between histamine-dependent and histamine-independent signaling pathways.

In 2006 Sonkoly et al. first suggested the role of interleukin 31 (IL-31) in pruritus accompanying atopic dermatitis [1]. Since that time, a number of studies have been undertaken to assess the meaningfulness of IL-31 in pruritus observed among other inflammatory skin conditions, like lichen planus, primary localized cutaneous amyloidosis, primary cutaneous T cell lymphomas, or mastocytosis [2–5]. The relevant role of IL-31 in pruritus pathogenesis has been recently confirmed in the phase II clinical trial with nemolizumab, an anti-IL-31 A receptor monoclonal antibody, in atopic dermatitis patients, showing significant decrease of pruritus intensity in active treatment group when compared to placebo [6]. Subsequent phase III clinical trials are ongoing, recruiting patients not only with atopic dermatitis, but also with other pruritic conditions, like, e.g., prurigo nodularis. Entering into this research trend, in this special issue, L. Kulczycka-Siennicka et al. analyzed the level of IL-31 in autoimmune blistering skin diseases frequently accompanied by pruritus, such as dermatitis herpetiformis and bullous pemphigoid. Surprisingly, they found decreased serum levels of IL-31 in patients suffering from these both conditions compared with healthy volunteers. Based on this observation, it could be suggested that IL-31 might not be the key cytokine mediating pruritus in these conditions; however, next studies are needed to further elucidate this phenomenon.

Interesting results on the itch and pain stimuli processing were also demonstrated by the group of A. I. M. van Laarhoven et al., who investigated whether attention is drawn to the stimulus location in tonic pruritus and pain stimuli. Opposite to their primary hypothesis, they were unable to find any indication for spatial attention allocation towards somatosensory stimuli, despite tonic itch and pain interfering with task performance. Based on these observations they concluded that patients with chronic pruritus or pain may benefit from learning to disengage their attention away from pruritus or pain, focusing attention on the location of pruritus or pain aggravates symptoms.

Several papers published in this special issue focused on the clinical presentation of pruritus in various conditions. V. Vachiramon et al. analyzed the prevalence and clinical manifestations of pruritus in vitiligo patients. Although vitiligo is usually not considered as a pruritic conditions, they found in the group of more than 400 patients that as much as about one fifth of patients with vitiligo may suffer from pruritus, with the highest prevalence in patients with focal vitiligo. Importantly, pruritus often preceded development of skin lesions suggesting that this phenomenon might be related to ongoing inflammatory response leading to destruction of melanocytes in the skin. Many patients with vitiligo experiencing pruritus declared disturbances in daily activity and problems with sleep, suggesting that this symptom should not be overlooked in this group of patients.

Similar situation was also observed among pregnant women, as demonstrated by J. Szczęch et al. Among the cohort of almost 300 women; the point prevalence of pruritus was about 20%, while the prevalence of pruritus during the entire pregnancy was almost 40%. Importantly, in many cases the presence of pruritus could not be attributed to any underlying condition suggesting that, indeed, pruritus may only be due to pregnancy. Frequent location within the abdomen skin suggests that at least in some pregnant women pruritus may be a result of skin stretching and mechanical activation of pruriceptive cutaneous nerve endings. Whether pruritus in pregnancy is also evoked by other mechanisms needs to be elucidated in the future.

Recently, greater attention has also been put on the scalp, as this body area can be very pruritic in such conditions as psoriasis and seborrheic dermatitis. As demonstrated by Yosipovitch group [7], skin on the scalp is highly innervated and as such may be more vulnerable to pruritic stimuli. Some researchers even suggested that pruritus in scalp psoriasis may be responsible for recalcitrant skin lesions due to Koebner phenomenon resulting from scratching.

Last but not least, we would like to put the readers' attention on two other papers related to pruritus assessment and treatment. On one hand, due to a subjective nature of pruritus, its objective measurement in clinical practice still remains a challenge. On the other hand, assessing the efficacy of any treatment options in clinical trials requires a valid instruments that are used to evaluate improvement of pruritus intensity [8]. Frequently, the Visual Analogue Scale or the Numeric Rating Scale are used; however, it is often not sufficient and these scales should be supplemented with other instruments. A. Reich et al. demonstrated a validation study on the new 12-Item Pruritus Severity Scale, which has been used by this group previously in various studies on pruritic diseases. In a number of analyses they showed that this itch severity questionnaire shows strong internal consistency, good reproducibility, and convergent validity and demonstrates significant correlation with patients' quality of life. Based on these observations it could be stated that the 12-Item Pruritus Severity Scale may be potentially used in future clinical trials.

Finally, A. He et al. reviewed current data on aprepitant, a neurokinin 1 receptor antagonists, in the treatment of pruritus and discuss its mechanism of action and adverse effects. Since first reports on its potential activity in chronic pruritus [9, 10], a growing interest is observed regarding neurokinin 1 blockade as a potential promising antipruritic target. Novel derivates with longer half-life time, such as serlopitant or tradipitant are currently investigated in clinical trials.

Summarizing, we hope, that the current special issue on pruritus would bring new insights into pathogenesis of pruritus, its assessment, and treatment, We also hope that the readers find the coverage interesting and important for their clinical practice.

*Adam Reich* *Laurent Misery* *Kenji Takamori*

## References

[1] Sonkoly E., Muller A., Lauerma A. I. (2006). IL-31: a new link between T cells and pruritus in atopic skin inflammation. *The Journal of Allergy and Clinical Immunology*.

[2] Welz-Kubiak K., Kobuszewska A., Reich A. (2015). IL-31 is overexpressed in lichen planus but its level does not correlate with pruritus severity. *Journal of Immunology Research*.

[3] Singer E. M., Shin D. B., Nattkemper L. A. (2013). IL-31 is produced by the malignant T-Cell population in cutaneous T-Cell lymphoma and correlates with CTCL Pruritus. *Journal of Investigative Dermatology*.

[4] Tey H. L., Cao T., Nattkemper L. A., Tan V. W. D., Pramono Z. A. D., Yosipovitch G. (2016). Pathophysiology of pruritus in primary localized cutaneous amyloidosis. *British Journal of Dermatology*.

[5] Lange M., Gleń J., Zabłotna M. (2017). Interleukin-31 polymorphisms and serum IL-31 level in patients with mastocytosis: Correlation with clinical presentation and pruritus. *Acta Dermato-Venereologica*.

[6] Ruzicka T., Hanifin J. M., Furue M. (2017). Anti-interleukin-31 receptor a antibody for atopic dermatitis. *The New England Journal of Medicine*.

[7] Bin Saif G. A., Ericson M. E., Yosipovitch G. (2011). The itchy scalp - scratching for an explanation. *Experimental Dermatology*.

[8] Ständer S., Augustin M., Reich A. (2013). Pruritus assessment in clinical trials: Consensus recommendations from the international forum for the study of itch (IFSI) special interest group scoring itch in clinical trials. *Acta Dermato-Venereologica*.

[9] Duval A., Dubertret L. (2009). Aprepitant as an antipruritic agent?. *The New England Journal of Medicine*.

[10] Ständer S., Siepmann D., Herrgott I., Sunderkötter C., Luger T. A. (2010). Targeting the neurokinin receptor 1 with aprepitant: A novel antipruritic strategy. *PLoS ONE*.

